# Validation of a System x_c_^–^ Functional Assay in Cultured Astrocytes and Nervous Tissue Samples

**DOI:** 10.3389/fncel.2021.815771

**Published:** 2022-01-13

**Authors:** Pauline Beckers, Olaya Lara, Ines Belo do Nascimento, Nathalie Desmet, Ann Massie, Emmanuel Hermans

**Affiliations:** ^1^Group of Neuropharmacology, Institute of Neuroscience, Université catholique de Louvain, Brussels, Belgium; ^2^Neuro-Aging & Viro-Immunotherapy, Center for Neurosciences, Vrije Universiteit Brussel, Brussels, Belgium

**Keywords:** xCT, synaptosomes, glutamate uptake, primary astrocyte culture, xCT knock out mice, cystine glutamate exchanger, SLC7A11 (xCT)

## Abstract

Disruption of the glutamatergic homeostasis is commonly observed in neurological diseases and has been frequently correlated with the altered expression and/or function of astrocytic high-affinity glutamate transporters. There is, however, a growing interest for the role of the cystine-glutamate exchanger system x_c_^–^ in controlling glutamate transmission. This exchanger is predominantly expressed in glial cells, especially in microglia and astrocytes, and its dysregulation has been documented in diverse neurological conditions. While most studies have focused on measuring the expression of its specific subunit xCT by RT-qPCR or by Western blotting, the activity of this exchanger in tissue samples remains poorly examined. Indeed, the reported use of sulfur- and carbon-radiolabeled cystine in uptake assays shows several drawbacks related to its short radioactive half-life and its relatively high cost. We here report on the elaborate validation of a method using tritiated glutamate as a substrate for the reversed transport mediated by system x_c_^–^. The uptake assay was validated in primary cultured astrocytes, in transfected cells as well as in crude synaptosomes obtained from fresh nervous tissue samples. Working in buffers containing defined concentrations of Na^+^, allowed us to differentiate the glutamate uptake supported by system x_c_^–^ or by high-affinity glutamate transporters, as confirmed by using selective pharmacological inhibitors. The specificity was further demonstrated in primary astrocyte cultures from transgenic mice lacking xCT or in cell lines where xCT expression was genetically induced or reduced. As such, this assay appears to be a robust and cost-efficient solution to investigate the activity of this exchanger in physiological and pathological conditions. It also provides a reliable tool for the screening and characterization of new system x_c_^–^ inhibitors which have been frequently cited as valuable drugs for nervous disorders and cancer.

## Introduction

Initially proposed in the early fifties, the role of glutamate as a neurotransmitter was demonstrated 20 years later. Since then, this rather simple amino acid has been unveiled as one of the most essential bioactive chemicals in the central nervous system (CNS) of mammalians. In most excitatory synapses, glutamate is released from presynaptic nerve terminals to activate a large diversity of ionotropic (iGluRs) and metabotropic (mGluRs) glutamate receptors. As major excitatory neurotransmitter, glutamate plays a crucial role in complex physiological processes including synaptic plasticity, learning and memory (Malenka and Nicoll, 1999). Glutamate also participates in the formation of glutathione (GSH), the most abundant antioxidant in the CNS (Had-Aissouni, 2012) and may serve as an alternative source of energy for diverse cells of the nervous system (McKenna, 2013). Besides these roles in physiological activities in the CNS, glutamate is also known for its implication in a large variety of nervous disorders (Lewerenz and Maher, 2015). High concentrations of this excitatory transmitter can cause damage to neuronal cells, a process known as excitotoxicity (Dong et al., 2009).

The control of glutamate transmission in the CNS does not solely depend on the interplay between neuronal cells, but also on closely associated glial cells in what is known as the tripartite synapse (Perea et al., 2009). Indeed, astrocytes play multiple roles in the control of glutamate homeostasis, in particular because they express excitatory amino acid transporters (EAATs), that ensure the clearance of extracellular glutamate and thereby protect neurons against excitotoxic insults (Anderson and Swanson, 2000; Oliet et al., 2001). Hence, impaired glial function and the associated excitotoxicity is frequently proposed as an important mechanism involved in several neurological disorders (Beart and O’Shea, 2007) and this has been validated in animal models of human diseases. Besides, astrocytes contribute to synaptic activity through the non-vesicular release of substantial amounts of glutamate by the cystine-glutamate exchanger, also known as system x_c_^–^ (De Bundel et al., 2011; Massie et al., 2011). Its widespread distribution throughout the CNS and the critical roles played by this exchanger suggest that it could be an important determinant of brain functions under physiological and pathological conditions (Lewerenz et al., 2013; Lutgen et al., 2014). There is a growing interest in the study of its physiological regulation in glial cells and in the possibility to develop specific drugs to pharmacologically manipulate its activity.

The cystine-glutamate exchanger is a Na^+^-independent transporter that is constituted of a heavy chain subunit common to several amino acid transporters, 4F2hc (encoded by the Slc3a2 gene), and a specific light chain subunit, xCT (encoded by the Slc7a11 gene). While the 4F2hc subunit ensures the trafficking and the cell surface expression of the heterodimer, xCT confers transport function and substrate specificity (Sato et al., 1999). Under physiological conditions, system x_c_^–^ supports the exchange of intracellular L-glutamate for extracellular L-cystine at a 1:1 molecular ratio, driven by their concentration gradients across the plasma membrane (Bannai, 1986). Once taken up by the cell, cystine is intracellularly reduced into cysteine, a building block for GSH synthesis which is essential to face oxidative stress (Bannai and Kitamura, 1980). Astrocytic GSH can be transferred to neurons and therefore, system x_c_^–^ contributes to the antioxidant protection throughout the CNS. Furthermore, through its glutamate release activity, the exchanger constitutes a substantial source of extracellular glutamate in several brain regions, likely impacting on the glutamatergic neurotransmission across the CNS (Barger and Basile, 2001; Massie et al., 2015).

The implication of system x_c_^–^ in essential functions of astrocytes has encouraged the study of its modulation in diverse conditions both *in vivo* and *in vitro*. Exposing cultured astrocytes to the pituitary adenylate cyclase-activating polypeptide (Kong et al., 2016), interleukin 1β (Jackman et al., 2010; Shi et al., 2016) or substance P (Johnson and Johnson, 1993) has been shown to regulate the exchanger. Besides, impaired expression or activity of system x_c_^–^ was documented in several animal models of neurological disorders such as Parkinson’s disease, amyotrophic lateral sclerosis and in glioblastoma (Lewerenz et al., 2013). So far, most of these studies have examined the regulation of system x_c_^–^ by RT-qPCR to quantify xCT mRNA. For a while, the lack of reliable anti-xCT antibodies has limited the study of its protein expression, yielding rather inconsistent published data (Van Liefferinge et al., 2016). Functional characterization of the cystine-glutamate exchanger is limited and frequently poorly described. In these studies, [^14^C] and [^35^S] radiolabeled-cystine is used as substrate in uptake assays (Bridges et al., 2001; Lewerenz et al., 2006; Liu et al., 2009; Massie et al., 2011; Merckx et al., 2015), but its high cost and relatively limited shelf-life constitute experimental drawbacks.

These issues have prompted us to validate a robust and cost-effective uptake assay for the functional study of system x_c_^–^ in physiological and pathological conditions. We here report the use of tritiated L-glutamate as a substrate for system x_c_^–^ (Bridges et al., 2001). Monitoring the reverse uptake of glutamate was already reported for measuring the activity of system x_c_^–^ on models of cell cultures (Gochenauer and Robinson, 2001; Shih and Murphy, 2001; Shih et al., 2003; Patel et al., 2004; Webster et al., 2014). In the current study, the specificity of this assay was strongly consolidated by using cell lines with induced or repressed xCT expression as well as crude synaptosome preparations and primary cultures of astrocytes derived from transgenic mice lacking xCT. Furthermore, the use of selective pharmacological inhibitors allowed us to distinguish the uptake supported by system x_c_^–^ or by EAATs.

## Materials and Methods

### Animals and Ethics Statement

All experiments were conducted in strict accordance with the recommendation of the European commission and with the agreement of the Belgian Ministry of Agriculture (code number LA 1230618). The Ethical Committee of the Université catholique de Louvain (UCLouvain) for animal experiments specifically approved this study (code number 2019/UCL/MD/033). xCT^+/+^, xCT^+/–^, and xCT^–/–^ mice used in this study were high-generation descendants of the strain previously described by Sato et al. (2005).

One to three days old pups and adult female mice [wild-type or xCT transgenic with a C57BL/6 background (Sato et al., 2005)] and rats (Sprague Dawley) aged between 10 and 12 weeks were used and kept in groups of maximum six per cage. All animals were accommodated under standard laboratory conditions, receiving food and water *ad libitum*, and were housed in the animal facility at the UCLouvain (Brussels), in controlled light/dark cycle, temperature and humidity conditions. xCT transgenic mice were genotyped by PCR on genomic DNA extracted from a tail biopsy using specific primers for Slc7a11 gene in order to identify bands corresponding to wild-type (xCT^+/+^), homozygous (xCT^–/–^), or heterozygous (xCT^±^) genotypes (Sato et al., 2005; De Bundel et al., 2011).

### Primary Cultures of Cortical Astrocytes

Cortices from rats or mouse pups were collected at postnatal day 2 and mechanically dissociated. Meninges were carefully removed, and astrocytes were separated from other cell types using 30 and 60% Percoll gradient (GE Healthcare) and seeded into coated flasks. Cells were left to proliferate at 37°C in a humidified atmosphere containing 5% CO_2_ in Dulbecco’s Modified Eagle’s Medium (DMEM-GlutaMAX, Thermo Fisher Scientific) supplemented with 10% fetal bovine serum (FBS) (VWR), 100 μg/mL penicillin-streptomycin (Thermo Fisher Scientific), 2.5 μg/mL fungizone (Thermo Fisher Scientific), 50 μg/mL L-proline (Thermo Fisher Scientific), and 50 μM of b-mercaptoethanol (Gibco). The medium was renewed on day 7, and on day 14, astrocytes were collected by trypsinization (Trypsin-EDTA, Thermo Fisher Scientific) and seeded at 70,000 cells per well in six-well plates for Western blotting and into poly-L-lysine coated 24-well plates at 35,000 cells per well for uptake measurement. On day 16, serum concentration was reduced to 3% as culture medium was renewed. When indicated, a supplement of 250 μM N^6^,2′-O-dibutyryladenosine 3′,5′-cyclic monophosphate (dBcAMP) (Sigma-Aldrich) was added on day 16. For all the experiments, the astrocytes were used after 23 days in culture.

### CRISPR/Cas9-Induced xCT Deletion in C6 Astrocytoma Cells

Guide RNA targeting the mouse xCT sequence (5′CACCGTCATTACACATACATTCTGG3′) was cloned into the lentiCRISPRv2-PURO vector (Backx et al., 2021). Lentiviral transduction and clone selection were performed as previously described by Backx et al. (2021) with little modifications. Briefly, cells were seeded in a six-well plate at a density of 100,000 cells per well. After 4 h, cells were transduced with lentivirus-containing supernatant supplemented with protamine sulfate (10 μg/mL, Sigma-Aldrich) and 50 μM b-mercaptoethanol for 24 h. Before renewing the medium (DMEM medium supplemented with 10% FBS, 100 μg/mL penicillin-streptomycin, 2.5 μg/mL fungizone, and 50 μM b-mercaptoethanol), cells were washed with PBS. After expanding the cells to T25 flasks and proliferation for 1 week, cells were selected with 5 μg/mL puromycin (InvivoGen) for at least 1 more week before any experiments were performed.

### Overexpression of xCT in Transfected Cells

The cDNA sequence encoding xCT (Slc7a11) was amplified from primary cultures of rat astrocytes and cloned into pcDNA3.1 (Invitrogen). Chinese hamster ovary (CHO) cells were cultivated at 37°C in a humidified environment (5% CO_2_) with Ham’s F-12 medium supplemented with 10% FBS and 100 μg/mL penicillin-streptomycin (Thermo Fisher Scientific). 2 × 10^6^ cells were seeded in a six-well plate a few hours before transfection with a mix consisting of 200 μL Ham’s F-12 medium, xCT DNA and X-tremeGENE™ HP DNA transfection reagent according to the manufacturer’s instructions. Selection with geneticin (G418) (1000 μg/mL, Gibco) was initiated 72 h after transfection, resulting in a stable transfected cell line, herein referred to as CHO-xCT. These cells were routinely passaged and grown in the presence of geneticin (250 μg/mL), except when plated for dedicated experiments.

### Crude Synaptosome Preparation

Mice were euthanized with CO_2_ and the spinal cord was immediately flushed out with phosphate-buffered saline (PBS; Meikle and Martin, 1981). Freshly isolated tissues were used for synaptosome preparation as previously described by Berger et al. (2011), with little modifications. Briefly, the samples were homogenized in an ice-cold solution of 320 mM sucrose by up-and-down movements with a pre-chilled Teflon/glass potter followed by up-and-down movements with a tight Downs. The homogenates were centrifuged at 1,000 *g* for 10 min at 4°C and the supernatants were carefully collected and stored on ice. The pellets were resuspended in ice-cold 320 mM sucrose solution and centrifuged again at 4°C at 1,000 *g* for 10 min. The two supernatants were combined and finally centrifuged at 4°C for 30 min at 17,500 *g*. The final pellet containing the crude synaptosomes was suspended in 1 mL of ice-cold appropriate buffer (see below). Protein concentration was determined by the Bradford method with the Bio-Rad protein assay (Bio-Rad Laboratories) and samples were diluted to 33.33 μg/mL for uptake assay.

### [^3^H]-L-Glutamate and [^3^H]-D-Aspartate Uptake Assays

On crude synaptosome preparations: To assess the uptake, [^3^H]-L-glutamate and [^3^H]-D-aspartate (with specific activity of 48.6 Ci/mmol and 12.2 Ci/mmol, respectively, PerkinElmer) were used as substrates at tracing concentrations of 20 and 50 nM, respectively. In a total volume of 500 μL, 10 μg of protein from the synaptosome preparation was incubated with the substrate at 37°C in a 96-well Masterblock (Greiner Bio-one). The assay was performed in a Na^+^-free buffer containing 140 mM *N*-methyl-D-glucamine (NMDG, as a substitute for NaCl), 5.4 mM KCl, 2.5 mM CaCl_2_, 1 mM MgCl_2_, 0.4 mM KH_2_PO_4_, 10 mM HEPES and 5 mM D-glucose, pH 7.4 adjusted with HCl, or with a Na^+^-containing buffer composed of 120 mM NaCl, 4.8 mM KCl, 1.2 mM KH_2_PO_4_, 1.2 mM MgSO_4_, 120 mM NaCl, 1.3 mM CaCl_2_, 25 mM HEPES pH 7.4, and 6 mM D-glucose. When indicated, homocysteic acid (HCA, Sigma-Aldrich) and L-threo-3-hydroxyaspartic acid (LTHA, Tocris) were included in the assay. After 20 min incubation at 37°C, the suspension was filtered through a GF/B glass fiber filter adapted to a 96-well plate (UniFilter GF/B, PerkinElmer), and washed three times with the ice-cold Na^+^-free buffer. After drying overnight at room temperature, MicroScint 20 (PerkinElmer) was added to each well of the filter plate. Plates were shaken for 2 h and then counted with a TopCount^®^ NXT Microplate scintillation and luminescence counter (PerkinElmer). Results are expressed as cpm per μg of protein.

On cell cultures: Primary cultured astrocytes or CHO cells were grown on poly-L-lysine coated 24-well plates. Culture medium was removed and immediately replaced with preheated Na^+^-free or Na^+^-containing buffers. After a brief incubation of 10 min, the plate was placed on the surface of a 37°C water bath and the buffer was removed and replaced with the same buffer supplemented with either [^3^H]-L-glutamate or [^3^H]-D-aspartate at a final concentration of 20 and 50 nM, respectively. When indicated, specific inhibitors (see above) were added to the assay. After 20 min ([^3^H]-L-glutamate) or 10 min ([^3^H]-D-aspartate), the uptake was stopped by three rinses with ice-cold buffer and cells were lysed with 0.1 M NaOH. A fraction of the lysate was collected and the radioactivity content was measured using the liquid scintillation solution MicroScint 40 and the TopCount^®^ NXT Microplate scintillation and luminescence counter. Another fraction of the lysate was used for protein quantification using the Bradford method with the Bio-Rad protein assay. Results are expressed as pmol of radiolabeled substrate transported per min per mg of protein.

### Western Blotting

Cells seeded in six-well plates were rinsed with PBS and scraped in ice-cold lysis buffer (100 mM Tris, 150 mM NaCl, 1 mM ethylene diamine tetra acetic acid, 1% Triton-X100, 0.1% SDS, 1% deoxycholic acid sodium salt, 99% pH 7.4) while synaptosome samples where directly resuspended in 1 mL of the same lysis buffer after the last centrifugation. Protein concentration was determined by the Pierce™ BCA method (Thermo Fisher Scientific) and samples were diluted to a concentration of 1 μg/μL. After adding Western blot loading buffer to the samples (final concentrations: Tris–HCl 60 mM pH 6.8, glycerol 10%, sodium dodecyl sulfate 2%,β-mercaptoethanol 5%, and bromophenol blue 0.01%), proteins were separated through a 10% SDS-PAGE and transferred to nitrocellulose membrane by electroblotting. Membranes were then incubated for 1 h in Tris-buffered saline (50 mM Tris pH 7.4, 150 mM NaCl) containing 0.05% Tween-20 and 5% bovine serum albumin (BSA, Carl Roth) to reduce non-specific labeling. Immunoprobing was carried out by incubating membranes overnight at 4°C with primary antibodies recognizing xCT [rabbit polyclonal antibody, 1:1000, (Massie et al., 2008; Van Liefferinge et al., 2016)] or EEF2 (rabbit monoclonal antibody, 1:1000, Thermo Fisher Scientific). Membranes were rinsed in TBS-Tween 0.05% and then incubated for 1 h with a peroxidase-conjugated secondary antibody (goat anti-rabbit IgG, 1:5000, Jackson ImmunoResearch Laboratories, Inc.). Immunoreactive proteins were detected with enhanced chemiluminescence reagent (Clarity, Bio-Rad Laboratories). Densitometric analysis of the signal was performed using ImageJ (Broken Symmetry Software).

### Statistical Analysis

Data were presented as means of three different experiments with the standard error of the mean (SEM). All statistical analyses were performed using GraphPad Prism version 5.01 (GraphPad Software, CA, United States). For multiple comparisons, data from distinct experimental conditions were analyzed using a one-way ANOVA followed by a Dunnett’s or a Bonferroni test. A *p*-value < 0.05 was considered significant for all statistical analyses.

## Results

### System x_c_^–^ and Excitatory Amino Acid Transporter-Dependent Uptake in Cultured Astrocytes and C6 Astrocytoma Cells

Primary cultures of astrocytes derived from the cortices of rat and mouse pups were used to measure the uptake of [^3^H]-L-glutamate and [^3^H]-D-aspartate. Indeed, astrocytes are best known for their capacity to take up excitatory amino acids through diverse EAATs which recognize both glutamate and aspartate whereas system x_c_^–^ only achieves the transport of glutamate. Another noticeable feature of EAATs is their dependency on the Na^+^ gradient across the cell membrane that provides the driving force for the uptake. Thus, in contrast to system x_c_^–^ of which activity is Na^+^-independent, EAAT activity requires the presence of a high concentration of Na^+^ in the buffer. To distinguish the [^3^H]-L-glutamate uptake supported by EAATs or by system x_c_^–^, the assays were therefore performed both in Na^+^-containing or Na^+^-free buffers.

**FIGURE 1:**
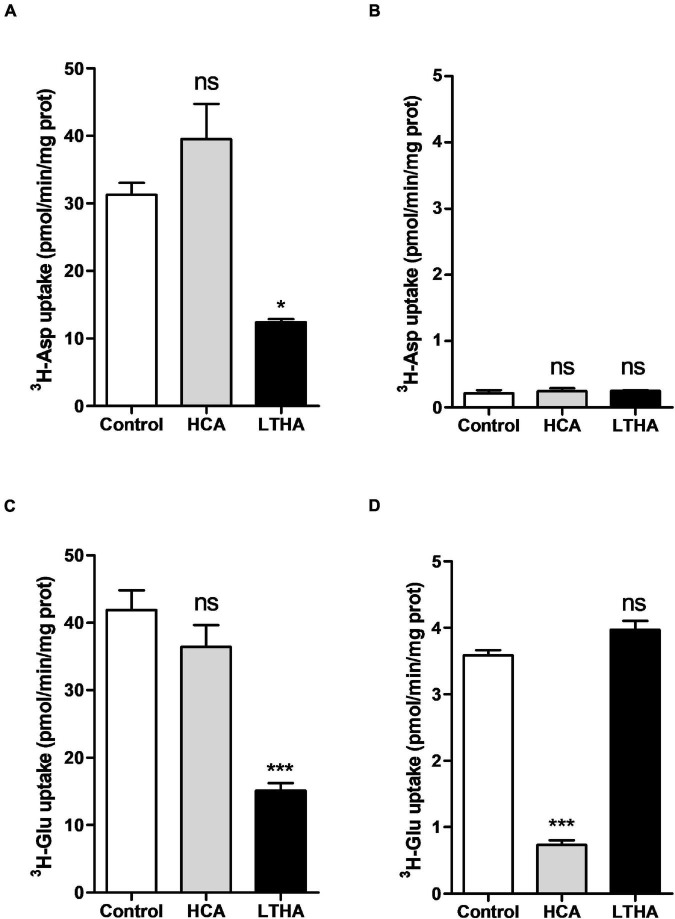
Influence of extracellular Na^+^ on [^3^H]-D-aspartate and [^3^H]-L-glutamate uptake in primary cultured rat astrocytes. The [^3^H]-D-aspartate **(A,B)** or [^3^H]-L-glutamate **(C,D)** uptake assays were performed in Na^+^-containing **(A,C)** or Na^+^-free **(B,D)** buffers. HCA (10^–4^ M) and LTHA (10^–4^ M) were used to discriminate the involvement of EAATs or system x_c_^–^ in the uptake. Data shown are the mean with SEM of the uptake capacity from three independent experiments performed in quadruplicate. Statistical analyses were performed using a one-way ANOVA followed by a Dunnett’s test for multiple comparisons (ns *p* > 0.05, *p < 0.05, ***p < 0.001 when compared to control).

As shown in Figures 1A,C, both substrates were taken up by rat derived astrocytes in the buffer containing Na^+^. On the other hand, only [^3^H]-L-glutamate was taken up in the Na^+^-free buffer (Figures 1B,D). The implication of EAATs and system x_c_^–^ in the uptake of the two substrates was further examined using specific inhibitors of these transporters. The system x_c_^–^ inhibitor HCA did not affect the uptake of [^3^H]-D-aspartate whereas a large proportion of this uptake was inhibited by LTHA (Figure 1A), an EAATs inhibitor. Regarding the influence of these inhibitors on the transport of [^3^H]-L-glutamate, data presented in Figure 1C showed that LTHA considerably reduced the uptake in the Na^+^-containing buffer whereas only HCA was inhibiting the uptake in the Na^+^-free buffer (Figure 1D). Together, these data confirm that astrocytes achieve glutamate uptake through both system x_c_^–^ and EAATs. Comparing the absolute uptake values in the different conditions indicates that in the Na^+^-containing buffer, EAATs predominantly contribute to the uptake of [^3^H]-L-glutamate that reaches up to 40 pmol/min/mg of protein. Conducting the assay with [^3^H]-L-glutamate in a Na^+^-free buffer appears as the optimal experimental condition to characterize the activity of system x_c_^–^ without any interference of EAATs. The uptake through system x_c_^–^ represents approximately one tenth of the total uptake in rat astrocytes (approx. 3.5 pmol/min/mg of protein).

**FIGURE 2:**
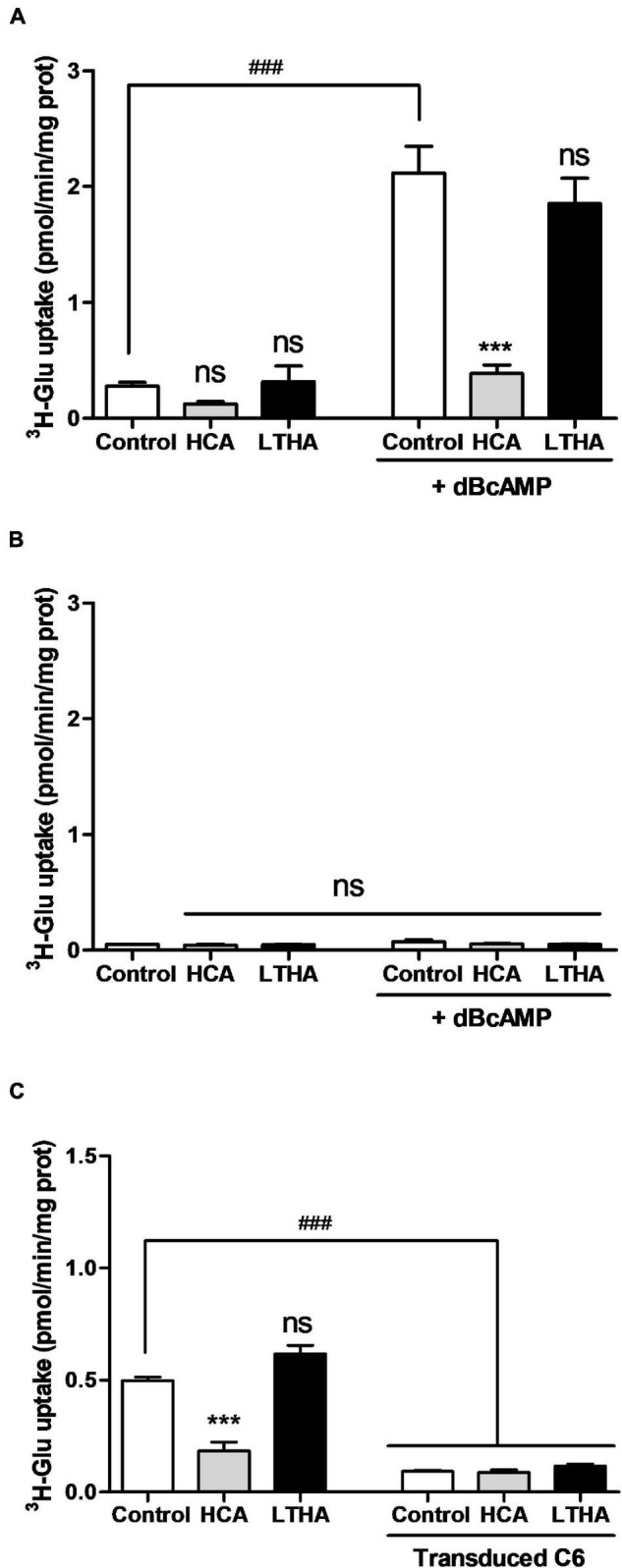
Influence of genetic xCT deletion on [^3^H]-L-glutamate uptake supported by system x_c_^–^ in cultured astrocytes and C6 astrocytoma cells. [^3^H]-L-glutamate uptake was quantified in primary cultured astrocytes derived from wild-type **(A)** or xCT^–/–^ mice **(B)** maturated for 7 days in the presence or the absence of dBcAMP, as well as on C6 astrocytoma cells with and without CRISPR/Cas9-mediated xCT deletion **(C)**. Specific pharmacological inhibitors were used (10^–4^ M) and added to the radioactive preparation when indicated. Histograms represent the mean with SEM of the uptake capacity from three independent experiments performed in quadruplicate. Statistical analyses were performed with a one-way ANOVA followed by a Dunnett’s test for multiple comparisons [ns *p* > 0.05, ***p < 0.001 refer to the comparison within the same cellular population (vs. control). ^###^*p* < 0.001 in **(A,B)** refers to the comparison between dBcAMP-maturated and non-maturated astrocytes or in **(C)** between C6 cells with and without xCT deletion].

The [^3^H]-L-glutamate uptake assay evaluating system x_c_^–^ activity was also performed in primary cultures of astrocytes derived from the cortices of xCT^+/+^ and xCT^–/–^ mouse pups. In astrocytes derived from xCT^+/+^ mice, the uptake of [^3^H]-L-glutamate measured in the Na^+^-free buffer was particularly low (Figure 2A). Exposing the cells during the period of maturation (7 days in a 3% FBS medium) to dBcAMP (250 μM) significantly increased the uptake values by 10-fold, reaching up to 2 pmol/min/mg of protein. Confirming the sole involvement of system x_c_^–^, this uptake was almost completely inhibited in the presence of HCA whereas LTHA was without effect. As shown in Figure 2B, the uptake of [^3^H]-L-glutamate was barely detectable in astrocytes derived from the transgenic mice lacking xCT, even when the culture was exposed to dBcAMP. Similarly, [^3^H]-L-glutamate uptake measured in the Na^+^-free buffer in the C6 astrocytoma cells was considerably reduced in the presence of HCA whereas LTHA was without such influence on the uptake capacity (Figure 2C). In the C6 cell line where xCT expression was specifically targeted using CRISPR-Cas9, the uptake of [^3^H]-L-glutamate was hardly detectable and was insensitive to HCA (Figure 2C). Together, these data validate the measure of [^3^H]-L-glutamate uptake in the Na^+^-free buffer as a selective functional monitoring of system x_c_^–^ activity.

### xCT Expression in Chinese Hamster Ovary Cell Increases Glutamate Transport Supported by System x_c_^–^

**FIGURE 3:**
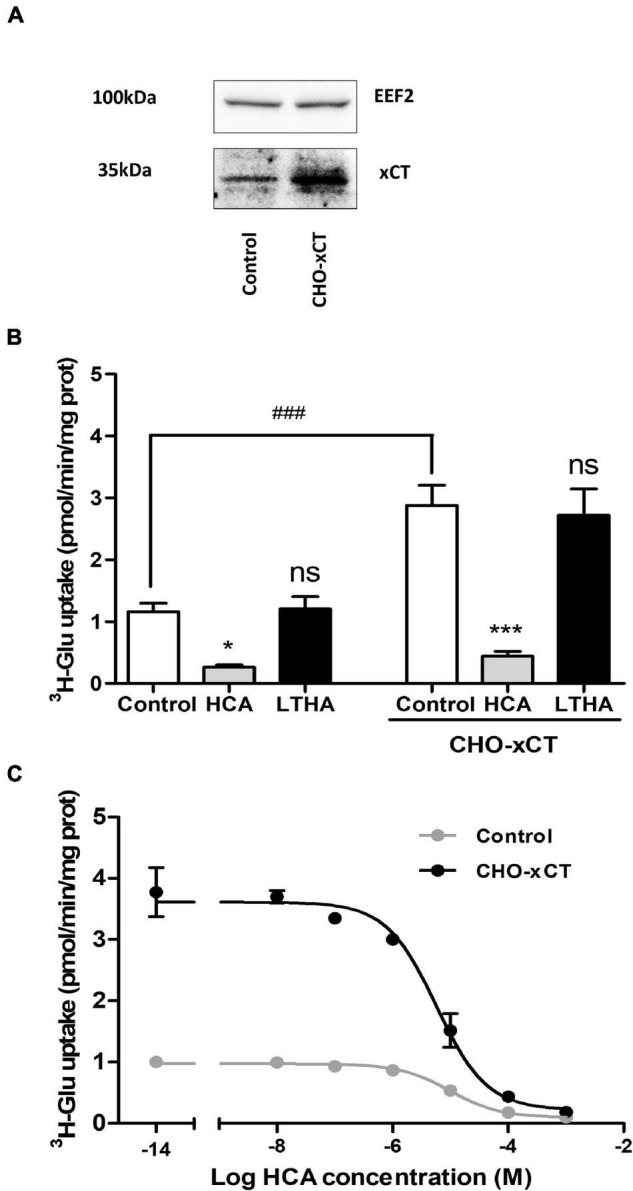
Influence of xCT overexpression on [^3^H]-L-glutamate uptake capacity of CHO cells. The increased expression of xCT was validated by Western blotting comparing protein samples from control and CHO-xCT cells using a specific rodent xCT antibody. Detection of the EEF2 was used as a control for sample loading and protein blotting. The blot shown is representative of three independent experiments **(A)**. [^3^H]-L-glutamate uptake capacity was measured on control and CHO-xCT cells. Pharmacological inhibitors (10^–4^ M) were added to the preparation to further confirm the nature of the transporter involved **(B)**. The potency of HCA at inhibiting system x_c_^–^-dependent [^3^H]-L-glutamate uptake capacity was examined by performing the assay in the presence of increasing concentrations of HCA **(C)**. Data represent the mean of the uptake capacity with SEM from three independent experiments performed in quadruplicate. Statistical analyses were performed using a one-way ANOVA followed by a Dunnett’s test for multiple comparisons [ns *p* > 0.05, *p < 0.05, ***p < 0.001 refer to the comparison within the same cellular population (vs. control). ^###^*p* < 0.001 refers to the comparison between CHO and CHO-xCT cells].

Chinese hamster ovary cells were stably transfected with the eukaryotic expression vector pcDNA3.1 carrying the rat xCT coding sequence. While some expression of xCT was detected by immunoblotting in samples from non-transfected cells, geneticin selected transfectants showed a higher expression level of the immunoreactive signal (Figure 3A). Accordingly, [^3^H]-L-glutamate uptake measured in the transfected cells was significantly higher as compared to non-transfected cells (Figure 3B). The specificity of this [^3^H]-L-glutamate uptake was confirmed using the system x_c_^–^ inhibitor HCA which causes a significant decrease in the uptake in both transfected and non-transfected cells. Non-linear analysis of the concentration-dependent uptake inhibition curves allowed to determine a half-maximal inhibitory concentration (IC_50_) of HCA in control and CHO-xCT cells in the 10 μM range (pIC_50_ values of 5.01 ± 0.12 and 5.24 ± 0.11, respectively) (Figure 3C). In contrast, LTHA did not influence the [^3^H]-L-glutamate uptake in these experimental conditions (Figure 3B).

### System x_c_^–^-Dependent Glutamate Uptake and xCT Expression in Crude Spinal Cord Synaptosomes

**FIGURE 4:**
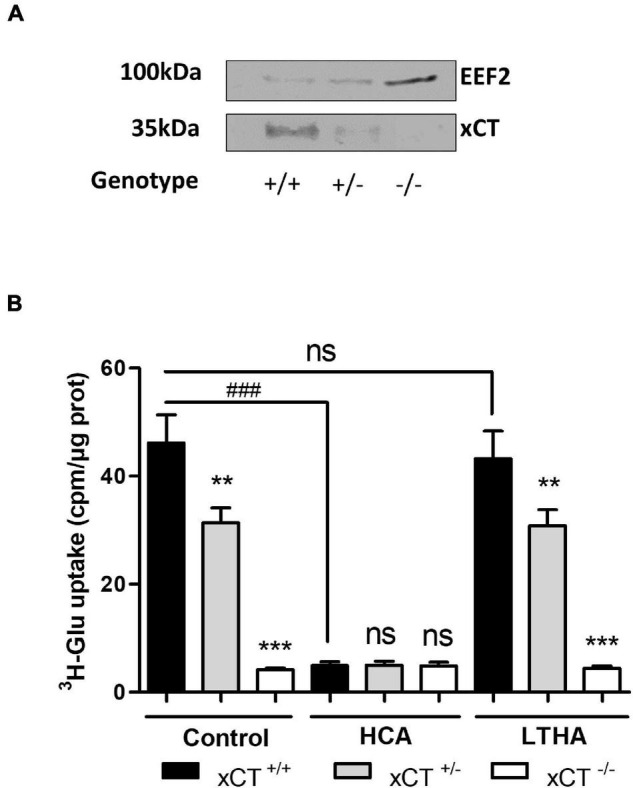
xCT expression and system x_c_^–^-dependent [^3^H]-L-glutamate uptake using spinal cord synaptosome preparations. xCT expression **(A)** and [^3^H]-L-glutamate uptake **(B)** in crude synaptosomes prepared from freshly isolated spinal cord samples. Tissues were obtained from transgenic xCT^+/–^ and xCT^–/–^ C57BL/6 mice and from wild-type littermates (xCT^+/+^). The genetic deletion of xCT was validated by Western blot using a specific antibody against rodent xCT. EEF2 detection was used as a control for sample loading and protein blotting. The blot shown is representative of three independent experiments. Histograms represent the mean with SEM of the uptake capacity from three independent experiments performed in quadruplicate. Statistical analyses were conducted using a one-way ANOVA followed by a Dunnett’s test for multiple comparisons [ns *p* > 0.05, **p < 0.01, ***p < 0.001 refer to the comparison between genotypes (vs. xCT^+/+^). ns *p* > 0.05, ^###^*p* < 0.001 refers to the comparison within the same genotype].

As previously described by Berger et al. (2011), crude synaptosomes from spinal cord samples were obtained after several steps of homogenization and centrifugation in ice-cold isotonic conditions. For the functional characterization of system x_c_^–^, crude synaptosomes from xCT^+/+^, xCT^+/–^, and xCT^–/–^ mice were prepared and resuspended in Na^+^-free buffer before the uptake experiment. The respective decreased expression or suppression of xCT in the heterozygous and xCT knockout animals was first validated by Western blotting on synaptosome samples submitted to detergent lysis (Figure 4A). In synaptosomes from xCT^+/+^ animals, a substantial [^3^H]-L-glutamate uptake was measured in the Na^+^-free buffer, which was almost completely inhibited in the presence of HCA (100 μM) but entirely preserved in the presence of LTHA (100 μM) (Figure 4B). Synaptosome samples prepared from the spinal cord of xCT^–/–^ mice presented a minimal glutamate uptake capacity as compared to xCT^+/+^ mice. This modest uptake was not affected by the pharmacological inhibitor HCA. In samples prepared from xCT^+/–^ mice, the substrate uptake value was estimated at 60% of the uptake measured in samples from the wild-type animals. In the conditions tested (Na^+^-free buffer), this uptake was completely inhibited in the presence of HCA and not influenced by LTHA.

## Discussion

While the implication of astrocytic EAATs in the control of glutamate homeostasis and the protection against excitotoxicity is well characterized (Kanai et al., 1993), accumulating reports identify system x_c_^–^ as another essential actor in the regulation of excitatory glutamate transmission in the CNS (Lewerenz et al., 2013; Williams and Featherstone, 2014; Bentea et al., 2020). By ensuring the uptake of cystine in exchange for glutamate, the activity of system x_c_^–^ provides astrocytes with cysteine that is incorporated into glutathione which serves as an antioxidant in the nervous tissues. This, however, concurs with the release of glutamate in the synapse.

While several studies have repeatedly documented the importance of EAATs dysfunction in the alteration of glutamate homeostasis (Rodríguez-Campuzano and Ortega, 2021), the impact of physiological and pathological changes affecting system x_c_^–^ should not be overlooked. So far, most studies have focused on assessing the expression of its specific subunit xCT by RT-qPCR or by Western blotting. Importantly, different studies have revealed an upregulation of xCT expression in animal models of several nervous system disorders, including Parkinson’s disease (Massie et al., 2008), epilepsy (Lewerenz et al., 2014), or amyotrophic lateral sclerosis (Mesci et al., 2015). This increased expression promotes the release of glutamate and it is indeed noteworthy that alteration in the glutamate homeostasis is commonly cited amongst the pathogenic mechanisms of these disorders [for review, see Massie et al. (2015)]. Beside these reports focusing on the regulation of xCT expression, only few studies have considered changes in the activity of the exchanger in neurological disease models. Functional studies are, however, essential in order to appreciate the consequences of the altered expression of the exchanger on the glutamate/cystine handling. Some laboratories have reported on *in vitro* and *ex vivo* experiments evaluating system x_c_^–^ activity using radiolabeled cystine ([^35^S]-cystine or [^14^C]-cystine) as substrate (Murphy et al., 1989; Fogal et al., 2007; Massie et al., 2011; Resch et al., 2014; Merckx et al., 2015). Previously validated on cell cultures, cystine uptake has been conducted on spinal cord and brain slices to monitor the activity of system x_c_^–^ (Kato et al., 1993; Melendez et al., 2005; Albano et al., 2013; Huang et al., 2018). This approach is, however, not routinely exploited as it presents several drawbacks including the high cost of these cystine radiochemicals and the limited radioactive half-life ([^35^S]-cystine). Siska et al. (2016) have recently reported on the use of a fluorescent derivative of cystine (fluorescein isothiocyanate – FITC) as a non-radioactive alternative to characterize system x_c_^–^ activity. The use of the cystine-FITC probe was validated on T-cells in flow cytometry, but its use for large scale pharmacological screening or functional studies on tissue samples requires further optimization. Moreover, the use of radiolabeled substrate in biochemical assays offers a higher sensitivity that is essential for quantitative studies on small biological samples.

For *in vitro* experiments, functional studies have been conducted using tritiated glutamate as substrate for a reverse transport supported by the exchanger (Gochenauer and Robinson, 2001; Shih and Murphy, 2001; Shih et al., 2003; Patel et al., 2004; Webster et al., 2014). Several transporters and ions channels, including system x_c_^–^ operate according to the concentration gradient of the transported chemicals across the cell membrane. For numerous transporters, a reversed activity has been documented in pathological conditions [GABA and glutamate transporters in epilepsy and ischemia (Szatkowski et al., 1990; Rossi et al., 2000; Wu et al., 2003)], in response to drugs [influence of psychostimulants on the dopamine transporter (Sulzer et al., 1995)] or at some stages of the development {chloride channel in the GABA_A_ ionotropic receptor [for review, see Peerboom and Wierenga (2021)]}. In biochemical assays, the transport direction can be manipulated by changing the extracellular concentrations of the transported chemicals and this is the case for system x_c_^–^ for which a reverse transport activity can be exploited. In the absence of extracellular cystine, the exchanger operates in a reverse direction and takes up glutamate (Bannai, 1986), which allows the use of radiolabeled glutamate to monitor system x_c_^–^ activity.

The use of glutamate as substrate for functional studies of system x_c_^–^, inevitably leads to difficult data interpretation related to the implication of other glutamate carriers. In particular, GLT-1 (EAAT2), which represents up to 1% of total proteins in the nervous system of mammals (Lehre and Danbolt, 1998). This transporter, commonly detected in maturated primary cultures of astrocytes, is recognized as the major contributor to glutamate uptake in most of the experimental models. The evaluation of the glutamate uptake specifically achieved by other mechanisms, including system x_c_^–^, necessitates to develop and validate experimental conditions that exclude the activity of GLT-1 and other EAATs. The strict Na^+^ dependency of EAAT, which is not shared by system x_c_^–^, offers the possibility to focus on the latter by removing Na^+^ from the assay buffers.

Even though a few publications have already exploited this approach (Bridges et al., 2001; Shih and Murphy, 2001; Shih et al., 2003; Patel et al., 2004; Melendez et al., 2005, 2016; Mysona et al., 2009), we here report on the validation of the assay and its selectivity using pharmacological inhibitors and genetically modified models, and its adaptation to samples of the nervous tissues where both glutamate transporters and the exchanger are expressed. Thus, in the absence of Na^+^, the uptake of radiolabeled glutamate was almost completely inhibited by HCA with an IC_50_ value consistent with a previous report on the inhibition of cystine uptake by system x_c_^–^ (Patel et al., 2004). In these conditions, the pan-EAAT inhibitor LTHA was without any significant impact on the glutamate uptake. Also, in the absence of Na^+^, the glutamate uptake measured in primary cultures of astrocytes derived from transgenic mice lacking xCT was barely detectable and unchanged in the presence of HCA. Together, these experimental observations validate the robustness and selectivity of the functional assay used for the evaluation of system x_c_^–^ activity.

Excitatory amino acid transporters and system x_c_^–^ also differ in their specificity for amino acid substrates. While EAATs equivalently transport the L- and D- enantiomers of both glutamate and aspartate, system x_c_^–^ only recognizes L-glutamate as substrate (Bridges et al., 2001; Patel et al., 2004). As a result, only EAAT-associated activity is assessed when examining the uptake of radiolabeled D-aspartate, as indicated here by the lack of its uptake in the absence of Na^+^. Quantitative analysis of the uptake values in these different experimental conditions allowed to clearly confirm that in primary cultured astrocytes, the glutamate uptake is predominantly assured by EAATs whereas system x_c_^–^ only partially contributes to the uptake (10%). Several protocols for the maturation/differentiation of cultured astrocytes derived from new-born rodent brain have been proposed (decrease in serum concentration, supplementation with a cocktail of growth factors or addition of dBcAMP) (Moonen et al., 1976; Sensenbrenner et al., 1980; Vermeiren et al., 2005; Prah et al., 2019). These procedures commonly increase the expression and activity of EAATs (Eng et al., 1997; Schlag et al., 1998; Vermeiren et al., 2005). Similarly, exposing the primary cultured astrocytes to dBcAMP during the differentiation step considerably increased the system x_c_^–^-dependent glutamate uptake, in accordance with the documented increased expression of the exchanger in the same conditions (Gochenauer and Robinson, 2001). The absence of dBcAMP-induced increase in Na^+^-independent glutamate uptake in astrocyte cultures derived from transgenic mice lacking xCT further consolidates the specificity of the assay.

The growing interest for the study of system x_c_^–^ as a major actor in the control of glutamate transmission is largely supported by reports highlighting the regulation of this exchanger in models of diseases both in peripheral tissues and in the CNS. While the characterization of system x_c_^–^ function in cell cultures may contribute to study some molecular mechanisms of its regulation, the possibility to examine the function of this exchanger in tissue samples is essential. We here successfully applied the reverse transport of radiolabeled glutamate to monitor the activity of system x_c_^–^ in crude synaptosomes prepared from the mouse spinal cord. These subcellular fractions are prepared from nervous tissues by homogenization and centrifugation in an isotonic buffer, yielding a suspension of large resealed cell vesicles with preserved membrane integrity and functional properties. The assay was adapted to a 96-well plate format allowing the harvesting of several samples simultaneously and limiting the amount of tissue needed to less than 10 μg of protein per sample. Initially performed on crude synaptosomes prepared from freshly dissected spinal cord, the experiment was successfully reproduced on hippocampal tissue as well as on frozen samples without loss of uptake activity (data not shown). Considering the heterogeneity of CNS samples and the large diversity of transporter systems in these tissues, the specificity of the assay appears essential. Our data evidence a substantial Na^+^-independent glutamate uptake in synaptosomes that was inhibited by HCA and totally absent in synaptosomes prepared from tissues of transgenic mice lacking xCT. Obviously, performing functional studies on synaptosomes appears far less quantitatively restrictive than on freshly prepared tissue slices, as frequently used for diverse transmitter uptake assays in the literature (Melendez et al., 2005; Albano et al., 2013). While an elaborated fractionation procedure can be used to enrich synaptosome preparations with nervous terminals, crude synaptosome samples are known to contain a large proportion of glial contaminants (Henn et al., 1976). As demonstrated for EAATs (Petr et al., 2015), system x_c_^–^ is predominantly expressed in glial cells but also detected in neurons (Jackman et al., 2010) and the possibility to discriminate the activity of the exchanger and other glutamate transporters is essential to study their specific regulation.

The documented increase in the expression of system x_c_^–^ in several neurological and non-neurological disorders, in particular, in contexts of inflammatory insults should encourage to consider this exchanger as a valuable pharmacological target. The protocol that we here describe and validate, appears as a robust, cost-effective, and reliable biochemical method for the screening and characterization of new system x_c_^–^ inhibitors both in cell culture models and in tissue samples. Combined with protein/mRNA expression studies it offers access to a comprehensive toolbox for the study of this exchanger in models of diseases.

## Data Availability Statement

The raw data supporting the conclusions of this article will be made available by the authors, without undue reservation.

## Ethics Statement

The animal study was reviewed and approved by the Ethical Committee of the Université catholique de Louvain (UCLouvain) for animal experiments. Written informed consent was obtained from the owners for the participation of their animals in this study.

## Author Contributions

EH and PB designed all the experiments and wrote the manuscript. PB and ND performed the experiments and analysis. AM helped for the experimental design and essential scientific feedback. AM and OL provided us with reagents and animals. IB assisted with the critical reviewing of the manuscript. All authors have read the manuscript and approved the submitted version.

## Conflict of Interest

The authors declare that the research was conducted in the absence of any commercial or financial relationships that could be construed as a potential conflict of interest.

## Publisher’s Note

All claims expressed in this article are solely those of the authors and do not necessarily represent those of their affiliated organizations, or those of the publisher, the editors and the reviewers. Any product that may be evaluated in this article, or claim that may be made by its manufacturer, is not guaranteed or endorsed by the publisher.
